# Intelligence

**Published:** 1810-06

**Authors:** 


### INTELLIGENCE.

Since the commencement of the present year, Hydrophobia has been extremely prevalent in France, particularly in the department of Paris. The following official notification on the subject has been inserted in all the French Journals: "The accidents caused by dogs having greatly increased for some time past, the Counsellor of State and Prefect of Police hereby inform such persons as shall in future be bitten by dogs supposed to be mad, that they ought to have recourse to a physician or surgeon within twenty-four hours; experience, which has not yet been contradicted by any authentic fact, having proved, that a deep cauterization of the bites made early with a red-hot iron, is a *certain method* of preventing the developement of hydrophobia. Persons who have been bitten, will find in all the hospitals of France, at all hours, the assistance which these accidents require. The Prefect of Police assures all persons that no reliance can be placed in any other remedy, whatever may be the confidence reposed in them by credulity or ignorance."

M. Parmentier has published some reflections on the Hypnum Cryspum, a species of moss, proposed, on account of the dearness of wool, as a substitute for stuffing mattrasses and furniture. The moss, which is of a moderate length, and has a somewhat fragrant smell, grows upon trees, particularly beech, is gathered in August and September, and when beaten like flocks, does not retain moisture or form into lumps like them. It is little liable to decay, and it is only necessary to dry it in the shade to preserve its fragrance. Neither sweat nor urine produces any fermentation in this moss, as it does in wool; but lest moisture should cause it to germinate, it may be steeped in lime water, which destroys its power of vegetation. It is said to be free from the property of imbibing and communicating contagion, which animal substances possess.

M. Ebel, of Bavaria, has recently published a geological work on the structure of the Alps, which is reported to contain much novelty, and to coincide entirely with the experiments made by Humboldt. According to their system, it is not true that granite is the nucleus of the surface of the earth; on the contrary, we find as many strata of granite as of any of the other integral substances of mountains. These strata of stones in the mountains were formed by crystalization in the sea of Chaos, and are found in a great measure on the same line from Savoy to Hungary. The earth, according to these ideas, resembles a prism of crystal, the edges of which have been worn away by the flux and reflux of the waters, without the ruins of these points having entirely filled up the cavities. This view of the subject is expected to lead to important results; but it will at the same time discourage those who still hope to find the solid nucleus of the earth. It begins to be embraced by the geologists of the continent, in preference to the systems which they had before adopted.

In our number for April, we noticed the recent disappearance of an island situated near the Cape of Good Hope, in consequence of an earthquake. The effects of this phenomenon at Cape Town, are detailed in the following letter from that place, begun to be written on December 6, 1809, and continued at different times:—On the 30th of November, the weather was unusually warm for so early a period of the season, the thermometer varying in the shade from 86° to 92°, with a sky perfectly clear and but little wind. Thus it continued till the evening of the 3d, when a cool westerly breeze, attended with a slight fog, came in from the sea. On the 4th, at nine A. M. the fog still continued; thermometer 74°, barometer 29° 80′. In the middle of the day, the mountains of Hottentot Holland, in the south-east, were covered with fleecy electric clouds, which are often observed at this time of the year. Several violent gusts of wind, which raised the dust to a considerable height in the air, were experienced in Cape Town, the intervals between them being perfectly calm. The sky for the whole day, after twelve at noon, except at Hottentot Holland, thirty miles from Cape Town, was perfectly clear. At five P. M. a strong south-east wind came on, unattended with the usual cloud over Table Mountain, which lasted three or four hours. At ten minutes past ten, P. M. a very violent shock of an earthquake was felt through the whole town, which was succeeded by two others equally tremendous; they continued about twelve or fourteen seconds, and followed each other at intervals of about half a minute, attended with a noise very different from thunder, but much louder. The shocks proceeded in the direction from south-east to north-west. Between the hours of ten at night of the 4th, and six in the morning of the 5th, about fourteen shocks were experienced; and two or three more in the course of the day. Excepting the first three, they were very slight; producing no perceptible motion of the earth, but resembling distant thunder. The last shock was at six A. M. this day (6th) but not stronger than the others. When the first shock was felt, the thermometer was at 77° in the house, probably at 74° out of doors. At two A. M. of the 5th, thermometer 68° in the open air; barometer at five P. M. on the same day, 29° 8′, wind west with rain; the night very dark. Next morning there was a very strong wind from the westward, and some rain. Several meteors, or falling stars, were observed during the night of the 4th, with a very luminous aurora australis. The ships in the bay, although the water was not apparently agitated, were so strongly affected by the shocks, that several men on board them were thrown out of their hammocks. I apprehend that nearly one-fourth of the houses in Cape Town are more or less damaged. Several pillars, urns, and other ornaments, have been destroyed. As yet I have heard of only one house that was entirely thrown down, but a great many have lost portions of their walls, and are cracked from top to bottom. The house which was demolished, was at some little distance from the town. The inhabitants in general forsook their houses during the whole whole night of the 4th, and so great was their consternation, that implicit credit was given to a very absurd prognostication, that similar shocks would be felt the next night. Of the Dutch inhabitants, I believe, not one went to bed before day-light. Tents were pitched in the parade, in the market, and in all the open places; and those who could not procure tents, had their waggons brought out and sat up in them. We have as yet received no particular accounts from the country; but innumerable vague reports are in circulation; and the inhabitants of the town, who are extremely susceptible of alarm, give credit to them all. One child of eight years old dropped down in the street, and instantly expired through terror. Two or three persons have been deprived of speech, and several others are suffering extremely in various ways, from the effect of extreme fear. Some are so much intimidated by this unexpected visitation, as seriously to talk of selling their houses and property here, and removing to Batavia. This powerful operation of terror on their minds, may probably appear astonishing to Europeans; but it is to be considered, that the inhabitants of this climate have been hitherto totally exempted from the tremendous convulsions of nature, which are frequently experienced in other quarters of the globe. December 7. We now find that the shocks, violent as they were, have not been felt at the hot baths, about eighty miles to the eastward, nor at sea, as we learn by the Camel, which ship arrived yesterday. It has been generally remarked, that a great many watches stopped, and several lost from two to ten, and even twelve and fifteen hours. Within the last half hour, we have had another slight shock. The inhabitants still continue in a considerable degree of alarm, and every unusual noise is dreaded as the forerunner of an earthquake. The following has been the state of the weather since the above-mentioned shock occurred:—

| Dec. | h. | m. | | Bar. | | Ther. | Wind. |
|---|---|---|---|---|---|---|---|
| 7 | 10 | 45 | P. M. | 30° | 20′ | 70 | S. W. |
| 8 | 2 | — | A. M. | — | — | — | —— |
| — | 5 | 30 | A. M. | — | — | — | —— |
| — | 3 | 10 | P. M. | 30° | 15′ | 73 | S. E. |
| 9 | 6 | — | P. M. | 30° | — | 73 | —— |
| 10 | — | — | —— | 29° | 75′ | 76 | —— |

No shock since the slight one of the 7th. Weather clear, except occasionally a fleecy cloud about the Table Mountain, aurora australis very strong at night, and many falling stars. It was remarked that animals, particularly horses, were much frightened at the shocks. Several moles are reported to have left their holes, and fled into the soldiers' tents at Wynberg, about seven miles from this place."

Mr. Wells's Gout Medicine was the juice of the berries of the Lacca Americana or Poke, preserved from fermentation by the addition of rum or brandy. The rich red colour induced the manufacturers and adulterators of wine to employ it for that purpose. Mr. Wells was convinced that it would flourish in any part of England land, and intended to furnish gentlemen with the means of propagating it in their own gardens, to which he said it would prove a considerable ornament.

Mr. CARPUE will commence his next Course of Lectures on Anatomy and Surgery, on Monday the 11th of June.—For particulars enquire at Mr. Carpue's House, Dean Street, Soho.

Dr. CLUTTERBUCK will begin his Summer Course of Lectures on the Theory and Practice of Physic, Materia Medica, and Chemistry, on Monday, June 4, at a quarter before ten in the morning, at his house, No. 1, Crescent, New Bridge Street.

Dr. REID will commence his Summer Course of Lectures on the Theory and Practice of Medicine, on Friday the 15th of June, at nine o'clock in the morning, at his house, Grenville Street, Brunswick Square.

Dr. GEORGE REES has a new Work in the Press, entitled, Practical Observations on Spasms of the Stomach and other morbid Affections of that Organ, with Remarks on the Use of the Bile in promoting Digestion.

Mr. PARKINSON has withdrawn the Introduction to the Knowledge of Fossils, announced at the end of his first volume of Organic Remains of a former World, considering its publication as entirely superseded by Mr. Martin's excellent systematic outlines of the same subject. The third edition of Organic Remains is in great forwardness.

Dr. STOCK's Life of Dr. Beddoes is in the press. It will comprise an analytical account of the Doctor's numerous writings, both published and unpublished.

The medical student and practitioner will soon receive from the pen of Dr. G. H. TOULMIN, of Wolverhampton, a work under the title of Elements of the Practice of Medicine, in which that important subject will, for the first time, assume all the interest of a practical science.

